# Short-term effects of antenatal betamethasone on fetal cardiovascular and circulation status: A quasi-experimental observational (before-after) study

**DOI:** 10.18502/ijrm.v22i5.16436

**Published:** 2024-07-08

**Authors:** Sedigheh Hantoushzadeh, Amir Amiri, Azadeh Shabani, Yasamin Soufi Enayati, Neda Mostafaeipour, Seyede Houra Mousavi Vahed, Maria Nezamnia, Toktam Sheykhian

**Affiliations:** ^1^Vali-E-Asr Reproductive Health Research Center, Family Health Research Institute, Imam Khomeini Hospital Complex, Tehran University of Medical Sciences, Tehran, Iran.; ^2^Maternal, Fetal, and Neonatal Research Center, Family Health Research Institute, Imam Khomeini Hospital Complex, Tehran University of Medical Sciences, Tehran, Iran.; ^3^Preventative Gynecology Research Center (PGRC), Shahid Beheshti University of Medical Sciences, Tehran, Iran.; ^4^Psychology Department, University of Science and Culture, Tehran, Iran.; ^5^Department of Obstetrics and Gynecology, Tehran University of Medical Sciences, Tehran, Iran.; ^6^Department of Obstetrics and Gynecology, Mashhad University of Medical Sciences, Mashhad, Iran.; ^7^Department of Gynecology, School of Medicine, Pasteur Hospital, Bam University of Medical Sciences, Bam, Iran.

**Keywords:** Betamethasone, Premature birth, Ultrasonography, Doppler, Echocardiography, Fetus.

## Abstract

**Background:**

The administration of antenatal corticosteroid is a standard treatment to reduce the rate of perinatal mortality and morbidity; however, there is limited evidence regarding the potential effects of betamethasone on the constriction of the ductus arteriosus (DA).

**Objective:**

This study aimed to investigate the short-term effects of antenatal betamethasone on fetal cardiovascular and circulation status.

**Materials and Methods:**

This quasi-experimental observational (before-after) study was conducted on 32 singleton fetuses. The participants were healthy pregnant women with a diagnosis of placenta accreta spectrum who were eligible for 2 doses of betamethasone and referred to prenatal care clinic, Vali-E-Asr hospital, Tehran, Iran from January 2021-May 2022. The results of fetal echocardiography and Doppler sonography were compared before and after the administration of antenatal corticosteroid therapy.

**Results:**

Following betamethasone injection, significant increases were observed in peak systolic and diastolic velocity of the DA without constriction of the DA (p < 0.001, p = 0.002 respectively). However, no significant changes were observed in right ventricular function, tricuspid valve function, Doppler of ductus venous, and peak systolic velocity of the aortic isthmus (p > 0.05). Doppler examination of the uterine, umbilical, and middle cerebral arteries also showed no significant changes (p > 0.05).

**Conclusion:**

Considering the benefits of antenatal corticosteroid therapy, its administration seems reasonable in preterm births. The transient changes in ductal blood flow are not prohibitive.

## 1. Introduction

Preterm birth is defined as birth occurring before 37 wk of gestation. The incidence of preterm birth ranges from 5–18% of all pregnancies (1–3). The administration of antenatal corticosteroids (ACS) is a standard treatment that has been shown to reduce the perinatal mortality and morbidity in these cases. However, there is limited evidence regarding the potential effects of betamethasone on the constriction of the ductus arteriosus (DA) due to its inhibition of prostaglandin synthesis (4–8).

DA is a vital structure in fetal circulation, carrying most right ventricular (RV) output to the descending aorta (9). Constriction of DA can lead to fetal pulmonary hypertension, tricuspid regurgitation (TR), and RV dysfunction, resulting in severe neonatal and fetal morbidity. The severity of its presentations may vary from mild symptoms to critical conditions like fetal hydrops (9, 10).

The aortic isthmus (AoI) represents a point of communication between the right and left fetal circulations. Changes in AoI blood flow may occur due to an imbalance between the vascular adaptations of the right and left heart circulations (11).

Previous studies have shown that the administration of more than 2 doses of betamethasone at gestational ages 26–27 wk can increase the risk of ductal constriction (12, 13). However, other investigations have demonstrated that the constricting effect of betamethasone on the DA is transient and time-dependent (14).

Considering the uncertainties regarding the side effects of a standard course of antenatal betamethasone on the DA, there is a strong need for further investigations.

Therefore, the present study aimed to examine the short-term effect of betamethasone administration on DA hemodynamics. Additionally, we assessed alterations in placental and fetal circulatory parameters. Moreover, we investigated whether changes in the DA Doppler parameters can affect the Doppler flow of AoI. Through this research, we hope to contribute valuable insights into the effects of betamethasone on fetal cardiovascular health and aid in better understanding its impact on the DA and other fetal circulatory parameters. Ultimately, this knowledge may assist in optimizing the use of ACS and improving the outcomes of preterm infants and their mothers.

## 2. Materials and Methods

This quasi-experimental observational (before-after) study was conducted on 32 singleton fetuses (of healthy pregnant women at risk of preterm labor and eligible for 2 doses of betamethasone) at the prenatal care clinic Vali-E-Asr hospital; Tehran, Iran, from January 2021-May 2022. 35 fetuses of healthy pregnant women with placenta accreta spectrum (PAS) entered the study, and 3 of them were excluded because of incomplete data.

The inclusion criteria were gestational age of 27–34 wk, singleton pregnancy with a diagnosis of placenta accreta spectrum, appropriate for gestational age, and normal prenatal screening results. Exclusion criteria were congenital anomalies based on ultrasound or echocardiography examinations, maternal systemic diseases, perinatal complications like intrauterine growth restriction (IUGR), and use of non-steroidal anti-inflammatory drugs and polyphenol-rich foods intake.

Betamethasone (Betamethasone sodium phosphate, Ampule, 4 mg/1 ml, Aburaihan Pharmaceutical Co., Iran) was administered as 2, 12 mg doses given deep injection intramuscularly, 24 hr apart (15).

Information regarding demographic and obstetric features of mothers and fetuses were gathered, encompassing details such as maternal age, weight, gestational age, the timing of ACS, and recorded fetal echocardiographic and sonographic parameters. A pediatric and fetal cardiologist carried out all echocardiographic examinations and an expert perinatologist performed all ultrasound examinations. Echocardiographic and ultrasound examinations were performed before administration of the drug and after the second dose.

The ultrasound and echocardiographic machine (Affinity 70 ultrasound system; Philips; Netherlands) with a C9–2 curved-array probe was used. Both cardiologists and perinatologists were blind regarding the study design.

The applied angle of insonation was < 20 . Through echocardiographic examinations, the peak systolic velocity (PSV; cm/s), peak diastolic velocity (PDV; cm/s), and the pulsatility index (PI) of DA were measured. The tricuspid annular plane systolic excursion (TAPSE; mm) and the PSV of the AoI were also measured to evaluate fetal cardiovascular status.

According to the literature, normal velocity through the AoI was a PSV of 120 cm/s or less throughout gestation age. The isthmic ﬂow index (IFI) was calculated by dividing the sum of the systolic (S) and diastolic (D) Doppler ﬂow integrals by the systolic ﬂow integral (IFI = S+D/S) (11).

**Figure 1:**
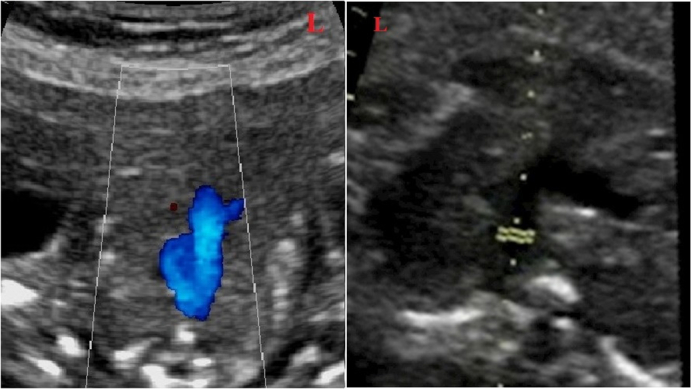
Color and pulsed Doppler examination of DA in the 3-vessel view.

Color Doppler examination of DA and AoI was performed in the 3-vessel view (Figure 1), and Doppler examination of ductus venosus (DV) was done in the sagittal view. The presence of a reversal wave at DV was also assessed. The tricuspid valve (TV) was evaluated immediately beyond the valve in the 4-chamber view. TAPSE was also recorded at the same view, placing the M-mode line at the lateral tricuspid valve annulus. The constriction of DA was considered by the presence of turbulent flow in the DA, with PSV greater than 140 cm/s, PDV greater than 35 cm/s, and PI < 1.9 (16).

Doppler sonographic examination of the middle cerebral artery (MCA), uterine artery (UtA), and fetal umbilical artery (UA) was performed to evaluate placental and fetal vascular hemodynamics. PSV and PI of the MCA, PI of uterine arteries, PI, and systolic/diastolic velocity ratio (S/D) of the UA were determined and recorded. For accurate measurement of MCA, color and pulse Doppler examinations were performed in the axial section of the brain. For evaluation of the UA and UtA flow, velocity waveforms were recorded at the middle free-floating part of the UA and uterine arteries at the apparent crossover of the uterine and external iliac arteries. Parameters were measured through 3 repeated cardiac cycles, and the mean values were calculated and recorded in a checklist.

### Sample size

Using the sample size formula to compare the means of variables (before/after) with considering a power of 90% and alpha error of 0.05, 35 participants entered the study as the minimum acceptable sample size. 


$$n\geq \frac{2{\left(Z_{1-\frac{\alpha }{2}}+Z_{1-\beta }\right)}^{2}}{({\frac{\delta_{\mathrm{𝐷𝑖𝑓𝑓𝑒𝑟𝑒𝑛𝑐𝑒}}}{\sigma_{\mathrm{𝐷𝑖𝑓𝑓𝑒𝑟𝑒𝑛𝑐𝑒}}})}^{2}}+\frac{{Z^{2}}_{1-\frac{\alpha }{2}}}{2}$$

### Ethical considerations

Informed consent was obtained from all pregnant women before enrolment. The project was approved by the Ethics Committee of the Tehran University of Medical Sciences, Tehran, Iran (Code: IR.TUMS.IKHC.REC.1399.353).

### Statistical analysis

All data were analyzed using the Statistical Package for the Social Sciences, version 23.0, SPSS Inc., Chicago, Illinois, USA (SPSS). Descriptive statistics for qualitative and quantitative variables were expressed in number (%) and mean ± SD. The relationships and differences between the before/after factors were assessed with a paired *t* test (all variables have a normal distribution). The p < 0.05 was considered significant.

## 3. Results

Of 35 included cases, 3 were excluded because of incomplete data, and 32 appropriate-for-gestational-age fetuses were entered into the study.

The means of maternal age and gestational age were 33.10 ± 4.995 yr and 31.19 ± 2.007 wk. The mean time between the echocardiographic examination and the first and second doses of betamethasone administration were 40 ± 3.00 and 15.00 ± 2.079 hr respectively. The detailed data are shown in table I.

**Figure 2:**
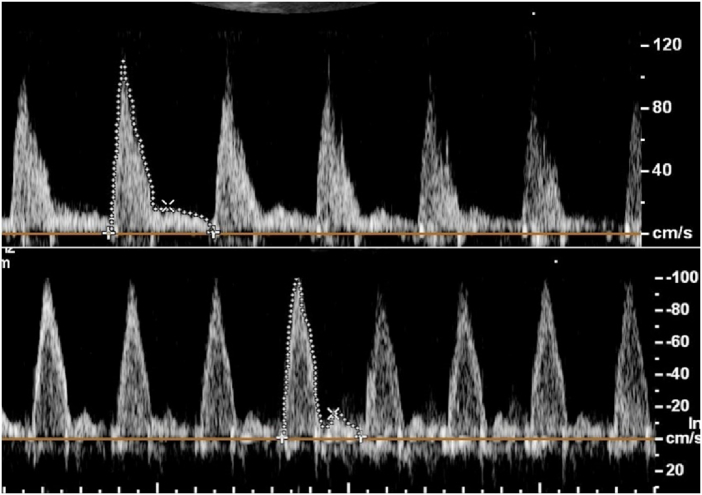
Doppler evaluation of the ductus arteriosus indicated higher peak systolic and diastolic velocities following the administration of betamethasone. In this particular instance, the initial systolic and diastolic velocities were: 98 cm/s and 15.7 cm/s (lower row), which subsequently increased to 108 cm/s and 17.2 cm/s respectively, 40 hr post-betamethasone administration (Upper row).

Color Doppler findings showed no TR before and after betamethasone injection. Analyses of data demonstrated that betamethasone administration could significantly increase DA PSV (p < 0.001) and PDV (p = 0.002). The means of DA PSV and PDV after betamethasone injection were significantly increased compared to these values before the injection of betamethasone (Figure 2). Comparing DA PI and TAPSE parameters, before and after drug injection, the results showed no significant changes. Also, we did not find abnormal turbulent blood flow in the DA. The sum of these findings shows no constriction in the DA.

The PSV value of AoI did not significantly alter after antenatal betamethasone administration (Table II). The IFI value in all cases was > 1.

Moreover, there was no reversal flow in the DV or alteration in its Doppler pattern. No significant changes were observed in the pulsatility indices of MCA, UA, and UtA after betamethasone administration (p < 0.05). The S/D ratio of the UA and PSV of the MCA had no significant changes after the drug administration. Detailed data are shown in table III.

**Table 1:** Descriptive data related to demographic and obstetric characteristics


**Variables**	**Mean ± SD**	**Median**	**Minimum**	**Maximum**
**Maternal age (yr)**	33.10 ± 4.995	34.00	22	42
**Maternal weight (kg)**	79.80 ± 8.124	79.00	59	96
**Gestational age (wk)**	31.19 ± 2.007	32.00	27	34
**Time of echo after ACS therapy (hr)**	15.00 ± 2.079	15.00	10	18
Data presented as Mean ± SD, ACS: Antenatal corticosteroids				

**Table 2:** Comparison of blood flow values related to DA and aortic isthmus before and after antenatal betamethasone injection


	**Before**		**After**			
**Indices**	**Mean ± SD**	**Median (min-max)**	**Mean ± SD**	**Median (min-max)**	**Mean**
	**difference**	P-value*			
**DA PSV (cm/s)**	84.940 ± 10.826		86.7500 (66.00–105.00)	101.459 ± 12.033	102.0000 (74.00–120.00)	16.5187 ± 4.829	< 0.001
**DA PDV (cm/s)**	14.818 ± 2.287	14.650 (11.00–20.00)	15.637 ± 2.326	15.350 (11.30–21.00)	0.819 ± 1.358	0.002
**DA PI**	2.989 ± 0.437	3.085 (2.200–4.200)	2.928 ± 0.379	2.900 (2.200–4.00)	-0.061 ± 0.305	0.266
**TAPSE (mm)**	5.730 ± 0.936	5.500 (4.40–8.00)	5.756 ± 0.899	5.500 (4.30–7.90)	-0.026 ± 0.251	0.566
**Isthmus PSV (cm/s)**	62.61 ± 12.310	64.00 (38.00–88.00)	63.625 ± 10.496	64.500 (41.00–88.00)	1.015 ± 4.074	0.169
*Data presented as paired *t* test, DA: Ductus arteriosus, PSV: Peak systolic velocity, PDV: Peak diastolic velocity, PI: Pulsatility index, TAPSE: Tricuspid annular plane systolic excursion						

**Table 3:** Comparison of blood flow values related to middle cerebral, umbilical, and uterine arteries before and after antenatal betamethasone therapy


	**Before**		**After**			
**Indices**	**Mean ± SD**	**Median (min-max)**	**Mean ± SD**	**Median (min-max)**	**Mean**
	**difference**	P-value*			
**MCA PSV (cm/s)**	40.830 ± 12.365		38.350 (25.00–64.00)	40.113 ± 11.334	38.000 (21.00–65.00)	-0.717 ± 1.031	0.653
**MCA PI**	2.034 ± 0.510	2.100 (1.18–3.70)	1.862 ± 0.401	1.905 (1.07–2.70)	-0.172 ± 0.109	0.126
**UA PI**	0.992 ± 0.255	0.990 (0.320–1.950)	0.985 ± 0.222	0.980 (0.690–1.80)	-0.007 ± 0.033	0.732
**UA S/D **	2.729 ± 0.669	2.600 (1.00–4.70)	2.517 ± 0.516	2.400 (1.80–3.90)	-0.212 ± 0.153	0.086
**UtA PI**	0.700 ± 0.222	0.660 (0.370–1.800)	0.692 ± 0.206	0.695 (0.360–1.00)	-0.008 ± 0.016	0.784
*Data presented as paired *t* test, MCA: Middle cerebral artery, PSV: Peak systolic velocity, PI: Pulsatility index, UA: Umbilical artery, S/D: Systolic/diastolic velocity ratio, UtA: Uterine artery						

## 4. Discussion

ACS therapy is commonly used for expectant mothers with preterm labor to promote fetal lung development. In this study, we assessed fetal cardiac metrics (DA, AoI, TAPSE, TR) and Doppler velocimetry of fetal and uteroplacental vessels (DV, UA, UtA, MCA) before and after betamethasone administration.

The results of our study indicated that peak systolic and diastolic gradients of the DA significantly increased after betamethasone administration. Nonetheless, the mean values of PSV and PDV of DA after betamethasone injection remained below the criteria for constriction of the DA. There was also a decrease in DA PI after betamethasone administration, although not statistically significant. Furthermore, no significant changes in parameters such as TR, TAPSE, and Doppler measurements of the DV were observed on a single course of betamethasone administration, in our participants. Despite the observed increases in DA gradients, no significant change was observed in the PSV of the AoI, indicating that the blood flow of the AoI was not affected by ACS therapy.

Our findings concur with 2 prior researches which demonstrated that the hemodynamic responses of the DA to the administration of betamethasone were mild. They concluded such effects contribute positively to the closure of the patent DA in premature infants (17, 18). Furthermore, one study proposed that antenatal betamethasone enhances the initial increase in pulmonary artery blood flow shortly after birth in preterm lambs. This initial rise is accompanied by a more pronounced redistribution of RV output toward the lungs (19). Nevertheless, opposing studies have suggested that the administration of corticosteroids to pregnant women with fetuses experiencing IUGR could elevate the risk of impaired RV function (20, 21).

Our further investigation revealed no notable changes in the values of the UtA after the administration of betamethasone. Although a minor decrease in the PI of the MCA and UA was observed, this decline did not attain statistical significance.

The existing literature presents conflicting findings concerning systemic vessel Doppler variations following the administration of steroids. Some studies are in alignment with us, while others are contradictory. For instance, a study suggested a potential vasodilatory impact of ACS, which led to a marked reduction in UA PI. Yet, the decrease in MCA PI was not statistically noteworthy (21). Another study demonstrated the absence of significant effects of betamethasone on both the fetal MCA and uteroplacental circulation (22). Similarly, Özdemir et al. identified no significant disparities in UA Doppler indices before and after steroid administration (23). In contrast to this study, one study stated that despite a significant initial decrease in MCA PI, these alterations were transient and maintained up to 72 hr (24). The results of an Iranian study showed that maternal antenatal betamethasone administration significantly decreased UA PI and significantly increased MCA PI (25).

The different findings in the existing literature revealed the variability in the effects of steroid administration on Doppler parameters of systemic vessels. This divergence may stem from variations in study methodologies, populations, or other factors.

The strength of our study lies in the homogeneity of the participants, as no cases of IUGR were included. Additionally, we conducted simultaneous examinations of both fetoplacental and cardiac circulation, which distinguished our study from previous ones that focused on only one aspect. Furthermore, we assessed the IFI, which allows us to visualize the blood flow of the descending aorta and the blood supply of the lower body. Notably, changes in the blood flow velocity waveform of the AoI occur earlier than those in the descending aorta, UA, and DV. This observation highlights the importance of AoI blood flow velocimetry as a potentially valuable critical tool in providing essential information about the fetal cardiovascular function (26).

However, our study has limitations, especially regarding the small sample size, particularly for cases with IUGR. For future investigations, we recommend larger studies that include a broader range of gestational ages, particularly extremely preterm cases and those with IUGR.

## 5. Conclusion

Our results revealed that ACS therapy does not appear to deteriorate the fetal condition, and nonsignificant changes in ductal blood flow are not prohibited with antenatal administration of betamethasone in preterm cases. Future studies with a larger sample size, including further extreme cases, are recommended.

##  Data availability

Data supporting the findings of this study are available upon reasonable request from the corresponding author.

##  Author contributions

Drafting of the manuscript: T. Sh., S. H., A. A., A. Sh., N. M., SH. MV., and M. N. Critical revision of the manuscript for important intellectual content: All authors. Statistical analysis: T. Sh., S. H., and Y. S. E. Supervision: T. Sh. and S. H.

##  Conflict of Interest

The authors declare that there is no conflict of interest.
